# Advances in the Understanding of Protein-Protein Interactions in Drug Metabolizing Enzymes through the Use of Biophysical Techniques

**DOI:** 10.3389/fphar.2017.00521

**Published:** 2017-08-08

**Authors:** Jed N. Lampe

**Affiliations:** Department of Pharmacology, Toxicology, and Therapeutics, University of Kansas Medical Center Kansas City, MO, United States

**Keywords:** cytochrome P450, protein-protein interaction, cytochrome P450 reductase, cytochrome b5, homodimer, heterodimer, allosterism, PGRMC

## Abstract

In recent years, a growing appreciation has developed for the importance of protein-protein interactions to modulate the function of drug metabolizing enzymes. Accompanied with this appreciation, new methods and technologies have been designed for analyzing protein-protein interactions both *in vitro* and *in vivo*. These technologies have been applied to several classes of drug metabolizing enzymes, including: cytochrome P450's (CYPs), monoamine oxidases (MAOs), UDP-glucuronosyltransferases (UGTs), glutathione S-transferases (GSTs), and sulfotransferases (SULTs). In this review, we offer a brief description and assessment of the impact of many of these technologies to the study of protein-protein interactions in drug disposition. The still expanding list of these techniques and assays has the potential to revolutionize our understanding of how these enzymes carry out their important functions *in vivo*.

## Introduction

Drug metabolism and disposition continues to be an important part of the drug discovery and development process in the pharmaceutical industry (Wienkers and Heath, 2005; Evers et al., 2013). While our knowledge of the structure-function relationships of the various drug metabolizing enzymes involved with metabolism and disposition has improved dramatically in the past 50 years (Ortiz de Montellano, 2005; Guengerich, 2006), there is still much that is unknown regarding their functioning *in vivo*. One particular aspect of this is how their structure and function may be modulated by protein-protein interactions.

Protein-protein interactions are a hallmark of biological systems. They mediate a vast number of critical cellular processes, including: cell division, hormone-receptor binding, signal transduction, enzyme allostery, molecular transport, and electron transfer (Murakami et al., 2017). Additionally, protein-protein interactions are essential to the function of many, if not all, of the enzymes involved in drug metabolism and disposition (Kandel and Lampe, 2014). These types of interactions may take the form of interactions with electron transfer partners, such as the case with cytochrome P450 (CYP) enzymes, or homo and hetero dimerization as observed with sulfotransferase (SULT; Yoshinari et al., 2001) and glutathione-*S*-transferase (GST; Balogh et al., 2009) enzymes. Protein-protein interactions between enzymes and proteins that are not directly required for enzymatic function may also be important for modulating specific activity of drug metabolizing enzymes in different contexts, such as the interaction of progesterone receptor membrane component 1 (PGRMC1) with CYP enzymes in the endoplasmic reticulum (ER) membrane (Rohe et al., 2009; also see the excellent recent review by Ryu et al., 2017). Unfortunately for the experimentalist, most protein-protein interactions are of a transient nature; i.e., short lifetime, low affinity, and low stability; which makes them difficult to analyze, since typically a large amount of stable complex is required for traditional biophysical techniques (Henzler-Wildman and Kern, 2007). This has hindered our basic understanding of the functional impact of many protein-protein interactions in drug metabolizing enzymes. Despite this, there have been numerous efforts made to apply biophysical methodologies and techniques to examine protein-protein interactions in proteins involved in metabolism and disposition. These range from traditional types of analysis, such as X-ray crystallography, NMR, and fluorescence, to more exotic techniques, such as luminescence resonance energy transfer (LRET) and conductometric monitoring. Each of these have their strengths and limitations in regards to the amount of sample needed, tolerance for lipid, cost, time commitment, and the type of observable information retrieved (Table 1). The reward for the experimentalist bold enough to apply these biophysical techniques is a richer understanding of protein-protein interactions in these important enzymes.

**Table 1:** A summary of the biophysical techniques discussed in this review and their application to monitoring protein-protein interactions in DMEs.

**Technique**	**Biophysical basis**	**Physical observables**	**Pros**	**Cons**	**Drug metabolizing enzyme (DME)**	**References**
X-ray crystallography	Electron diffraction by protein crystal lattice	Atomic bond position and length	Definitive structural assignment of all the residues in a polypeptide	Requires a large amount of protein and limited to a “snapshot” in time	CYP, CPR, GST, SULT, MAO, UGT, and others	Sevrioukova et al., 1997, 1999; Cupp-Vickery et al., 2000; Hall et al., 2001; Podust et al., 2001; Yoshinari et al., 2001; Williams et al., 2003; Scott et al., 2004; Yano et al., 2004; Nagano and Poulos, 2005; Nagano et al., 2005; Grahn et al., 2006; He et al., 2006; Hamdane et al., 2009; Deng et al., 2010; Vincent et al., 2012; Wilderman et al., 2012; Hiruma et al., 2013; Tripathi et al., 2013; Peng et al., 2014; Sugishima et al., 2014; Basudhar et al., 2015; Reed and Backes, 2017; Gill, 1983; Kakuta et al., 1997; Meech and Mackenzie, 1997; Argiriadi et al., 1999; Binda et al., 2001, 2011; Petrotchenko et al., 2001; Polekhina et al., 2001; Abdalla et al., 2002; Miley et al., 2007; Wang and Edmondson, 2007; Schoch et al., 2008; Lewis et al., 2011; Nelson et al., 2013; Matsumoto et al., 2014; Suzuki et al., 2014; Fujiwara et al., 2016; Audet-Delage et al., 2017; Chenge et al., 2017
NMR	Measures changes in magnetic resonance of atomic nuclei (typically ^1^H)	Provides detailed information about the structure, dynamics, reaction state, and chemical environment of macromolecules	Potential for complete structural assignment of the entire protein; also captures protein dynamics	Large amount of protein required; must be soluble	CYP, CPR (domains only), GST, and SULT	Lian, 1998; McCallum et al., 1999; Mahajan et al., 2006; Kijac et al., 2007; Lampe et al., 2008, 2010; Vallurupalli et al., 2008; Gluck et al., 2009; Raman et al., 2010; Ahuja et al., 2013; Estrada et al., 2013, 2014, 2016; Hiruma et al., 2013; Basudhar et al., 2015; Cook et al., 2016; Zhang et al., 2016
Fluorescence: FRET/BRET	Energy transfer from a donor fluorophore to an acceptor fluorophore through non-radiative dipole–dipole coupling	Intermolecular distance, detection of direct molecular interaction between two proteins	Unique information regarding protein dynamics and specific residues involved in the interaction	(Usually) requires the addition of an exogenous fluorophore	CYP, CPR, GST, UGT	Nisimoto et al., 1983; Schwarze et al., 1983; Wang et al., 1993, 2001; Davydov et al., 1996, 2000, 2010, 2013a,b, 2015; Dietze et al., 1996; Lakowicz, 1999; Lu and Atkins, 2004; Wen et al., 2006; Praporski et al., 2009; Li et al., 2011; Yuan et al., 2015, 2016; Fujiwara et al., 2016
Fluorescence: fluorescence anisotropy	Measurement of the photon emission of a fluorophore along different axis of polarization	Measurement of intermolecular binding constants and reaction kinetics	Allows monitoring of direct interaction in real time	Requires exogenous fluorophore with high quantum yield	CYP, GST	Greinert et al., 1979, 1982; Gut et al., 1985; Schwarz et al., 1993; Gorovits and Horowitz, 1995; Lim et al., 1995; Lakowicz, 1999; Szczesna-Skorupa et al., 2003
Photoaffinity labels/peptide MS	Covalent alkylation of a protein with a photo-reactive group, such as an azide, a diazirine, or benzophenone	Identification of specific sites of protein-protein interaction and distances	Provides direct information on specific sites of interaction	Requires introduction of an exogenous photolabile probe on the protein	CYP	Hodek and Smrcek, 1999; Wen et al., 2005; Gao et al., 2006a; Sulc et al., 2008
Chemical crosslinking/peptide MS	Covalent modification of a protein with a chemically reactive group, such as a malimide, iodoacetamide, or isothiocyanates	Identification of specific sites of protein-protein interaction and distances	Provides direct information on specific sites of interaction	Requires introduction of an exogenous chemically labile probe on the protein	CYP, GST	Cooper, 2002; Gao et al., 2006b; Losel et al., 2008; Reed et al., 2010
SPR/Surface immobilization	Determines the change in refractive index of incident light on a surface bilayer due to resonant oscillation of conduction electrons at the interface	Determination of binding constant (Kd) of interaction, thermodynamic analysis, epitope mapping	Allows for determination of binding constants and mode of interaction	Requires that one protein partner be immobilized on a chip surface	CYP	Ivanov et al., 1997, 1999, 2001; Kuznetsov et al., 2004; Shimada et al., 2005; Pearson et al., 2006; Archakov and Ivanov, 2011; Martin et al., 2015; Bostick et al., 2016; Yablokov et al., 2017
Quartz crystal microbalance conductometric monitoring	Conductometric biosensor coupled with *in vitro* transcription/translation system for monitoring protein-protein interactions using a quartz crystal microbalance with dissipation monitoring	Disassociation constants (Kd), thermodynamic parameters, complex size	Allows for determination of binding and thermodynamic constants	Requires efficient combined transcription and translation of protein	CYP	Davydov et al., 2013b; Spera et al., 2013

In this review, we offer an overview of many of the biophysical techniques that have been applied over the years to understand protein-protein interactions in drug metabolizing enzymes, with a focus on the techniques themselves and the salient information that has been obtained from each. Additionally, we will provide a perspective on the future of the field and some newly emerging techniques of interest to the active researcher.

## X-ray crystallography

Over the course of the previous two decades, X-ray crystallography has provided us with an enormous amount of information regarding protein structure in drug metabolizing enzymes (Cupp-Vickery et al., 2000; Podust et al., 2001; Yoshinari et al., 2001; Williams et al., 2003; Yano et al., 2004; Nagano and Poulos, 2005; Nagano et al., 2005; Grahn et al., 2006; He et al., 2006; Wilderman et al., 2012; Basudhar et al., 2015), for a recent review in this area, see (Reed and Backes, 2017). Despite this, structures of multi-protein complexes involved in drug metabolism and disposition are exceedingly rare. While direct examples of crystal structures of protein complexes are uncommon, there have been many clues provided as to how proteins might interact with one another in their native state. One such case is the interaction of CYPs with their endogenous electron transfer partners, cytochrome P450 reductase (CPR) and cytochrome *b*_5_ (*b*_5_). In order to oxidize the various drugs that serve as their substrates, CYPs must receive electron reducing equivalents from CPR and/or *b*_5_ (Ortiz de Montellano, 2005; Henderson et al., 2013). This involves formation of a protein complex that allows electrons to be directly shuttled from CPR/*b*_5_ to the CYP. Despite more than 50 years of study, the exact molecular process by which this occurs still remains somewhat of a mystery. X-ray crystal structures utilizing traditional techniques have demonstrated that the interaction is predominantly mediated by electrostatics (Hiruma et al., 2013; Tripathi et al., 2013). However, the question has remained as to the role of conformational dynamics in the electron transfer process, as the CPR enzyme has previously been crystalized in a “closed” conformation (Vincent et al., 2012) that limits access to the flavin mononucleotide (FMN) domain. In order to determine the distinct conformational state of CPR that interacts with the CYP enzyme, a co-crystal structure of the two proteins in complex was needed. A breakthrough came in this regard when Sevriukova and colleagues were able to determine the crystal structure between the heme and FMN-containing domains of the model cytochrome P450BM-3 (Sevrioukova et al., 1999). This was facilitated by a novel strategy of individual expression and purification of the heme, FMN, and FAD containing domains of the enzyme (Sevrioukova et al., 1997) and then carefully reconstituting both the heme containing domain with the FMN domain (Sevrioukova et al., 1997, 1999). The structure itself indicated that the proximal side of the heme was the primary site of interaction with the FMN domain and, furthermore, identified a pathway for electron transfer from the FMN domain to the heme iron (Sevrioukova et al., 1999). This led to the hypothesis, developed somewhat later, that a “hinging” motion opens the protein and allows the FMN domain to directly interact with the CYP heme (Hall et al., 2001; Hamdane et al., 2009; Sugishima et al., 2014).

A clever strategy involving crystallization of a four amino acid deletion mutant demonstrated that the CPR protein can pivot along the C terminus of the hinge region and thereby undergo a conformational rearrangement that allows sufficient opening of the protein to expose the FMN domain to the CYP interface (Hamdane et al., 2009). Interestingly, this hypothesis of a conformational rearrangement being necessary for an effective route of electron transfer to be formed between the CPR and CYP proteins was later confirmed when CPR was co-crystalized with heme oxygenase (HO; Sugishima et al., 2014). In this complex, it became clear that shortening the CPR hinge region leads to a protein that favors the open conformation and promotes association with HO (Sugishima et al., 2014). These studies demonstrate the utility of combining the power of X-ray crystallography with traditional site-directed mutagenesis techniques to obtain answers to structural questions that might not be readily available by either technique alone.

X-ray crystallography has also been useful for dissecting the protein-protein interactions of another CYP electron transfer partner, *b*_5_. Using a combination of chemical crosslinking and protein crystal structures, Peng et al. were able to propose a model for the *b*_5_ mediated stimulation of the 17,20-lyase activity of cytochrome P450c17 (Peng et al., 2014), suggesting that electrostatic interactions predominate in the CYP-b5 interaction, as they do with the CYP-CPR complex. In another example, X-ray crystal structures of *b*_5_ have informed our understanding of electron transfer from *b*_5_ to individual CYPs, such as CYP2B4, where ligand-induced structural changes were found to be coupled to *b*_5_ effector binding (Scott et al., 2004).

**Figure 1:**
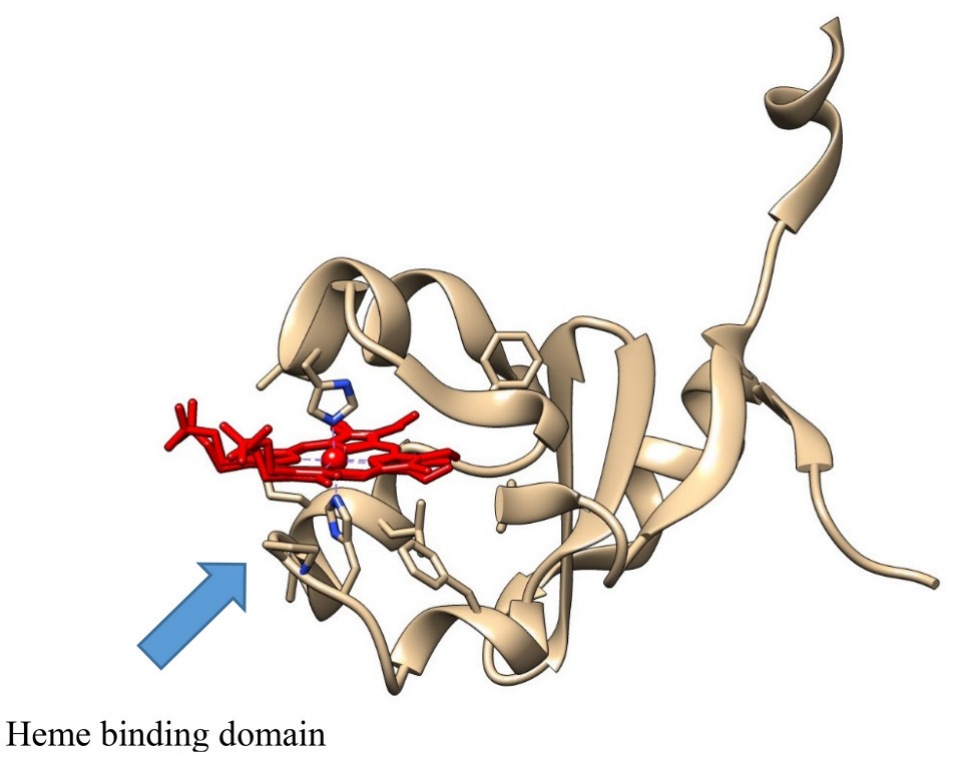
The crystal structure of the *b*_5_-like protein, Ncb5or (PDB: 3LF5), showing the heme (in red), with the Fe atom coordinated between the two active site histidine residues.

It has also been useful in defining novel interactions with CYP proteins, as well as those currently known. In an X-ray crystallography study comparing the *b*_5_-like protein, Ncb5or, with *b*_5_, Deng et al. determined that the positioning of the second histidine heme ligand (His 112) in *b*_5_ is critical for efficient electron transfer, both from *b*_5_ reductase and to the (CYP) electron transfer partner (Figure 1; Deng et al., 2010). These are just a few of the many examples available where X-ray crystallography has been used to interrogate CYP and electron transfer partner interactions.

However, CYPs are also known to form both hetero and homo oligomers which can influence their functional activity (Davydov, 2011, 2016; Reed and Backes, 2016, 2017). In this aspect, X-ray crystallography has contributed to our structural understanding of the factors involved in dimer formation. For example, the *M. tuberculosis* CYP126A1 protein was recently demonstrated to form homodimers under crystallization conditions (Chenge et al., 2017). The dimer interface is composed of interactions along the hydrophobic B/C and F/G loop regions of the protein, which are known substrate recognition regions. Interestingly, the CYP126A1 protein forms a dimer both in the ligand-free state and also with substrate bound. However, the dimer is disrupted when the inhibitor ketoconazole is bound to the protein, leading to conversion to the monomer (Chenge et al., 2017). Similarly, CYP2C8 has been shown to crystalize as a dimer (Schoch et al., 2008). As observed with CYP126A1, the CYP2C8 homodimer was formed around interactions at the hydrophobic F/G loop interface. The presence of the dimer was also confirmed in solution as well as under the original crystallization conditions used (Schoch et al., 2008). Remarkably, two molecules of the substrate palmitic acid were found to be bound in the dimer interface, illustrating the potential physiological relevance of dimer formation. In contrast to CYP126A1, the presence of ligand did not prevent dimer formation, suggesting that homodimeric protein-protein interaction modes may vary between different CYPs. Despite this, the peripheral ligand binding site identified in CYP2C8 has been proposed to be important in modulating the cooperative effects observed with multiple ligand binding in certain CYPs (Davydov et al., 2013a, 2015; Reed and Backes, 2017), and may also serve as a “hot spot” for protein-protein interaction.

**Figure 2:**
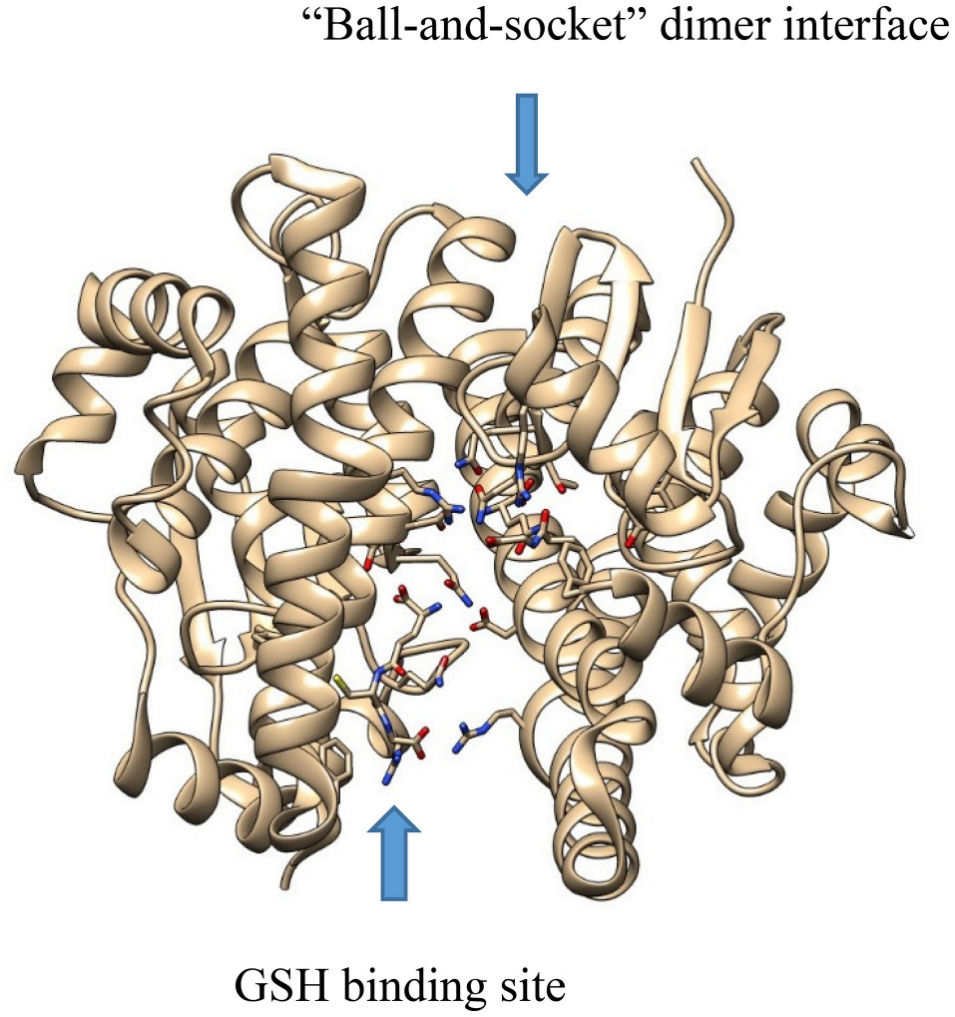
The crystal structure of GST A1-1 (PDB: 3I6A), showing the “ball-and-socket” dimer interface and the GSH binding site.

X-ray crystallography has also been useful in informing our understanding of the function of protein dimers of other drug metabolizing enzymes as well. Both cytosolic GST (Balogh et al., 2009) and SULT (Yoshinari et al., 2001) enzymes have been crystalized as dimers. Moreover, dimerization seems to be important to function in both classes of enzymes (Kakuta et al., 1997; Petrotchenko et al., 2001; Abdalla et al., 2002). In the case of GST enzymes, the dimer is composed of a two-fold axis between each monomer with multiple hydrophobic, so called “ball-and-socket”, interactions between the different domains of each monomer (Figure 2) (Balogh et al., 2009). The crystal structure of maleylacetoacetate isomerase/glutathione transferase zeta revealed that residues M51 and F52 from a loop between helix α2 and strand β3, where the residues are wedged into a hydrophobic pocket formed between the α4 and α5 helices, thereby vividly illustrating the ball-and-socket type structure (Polekhina et al., 2001).

**Figure 3:**
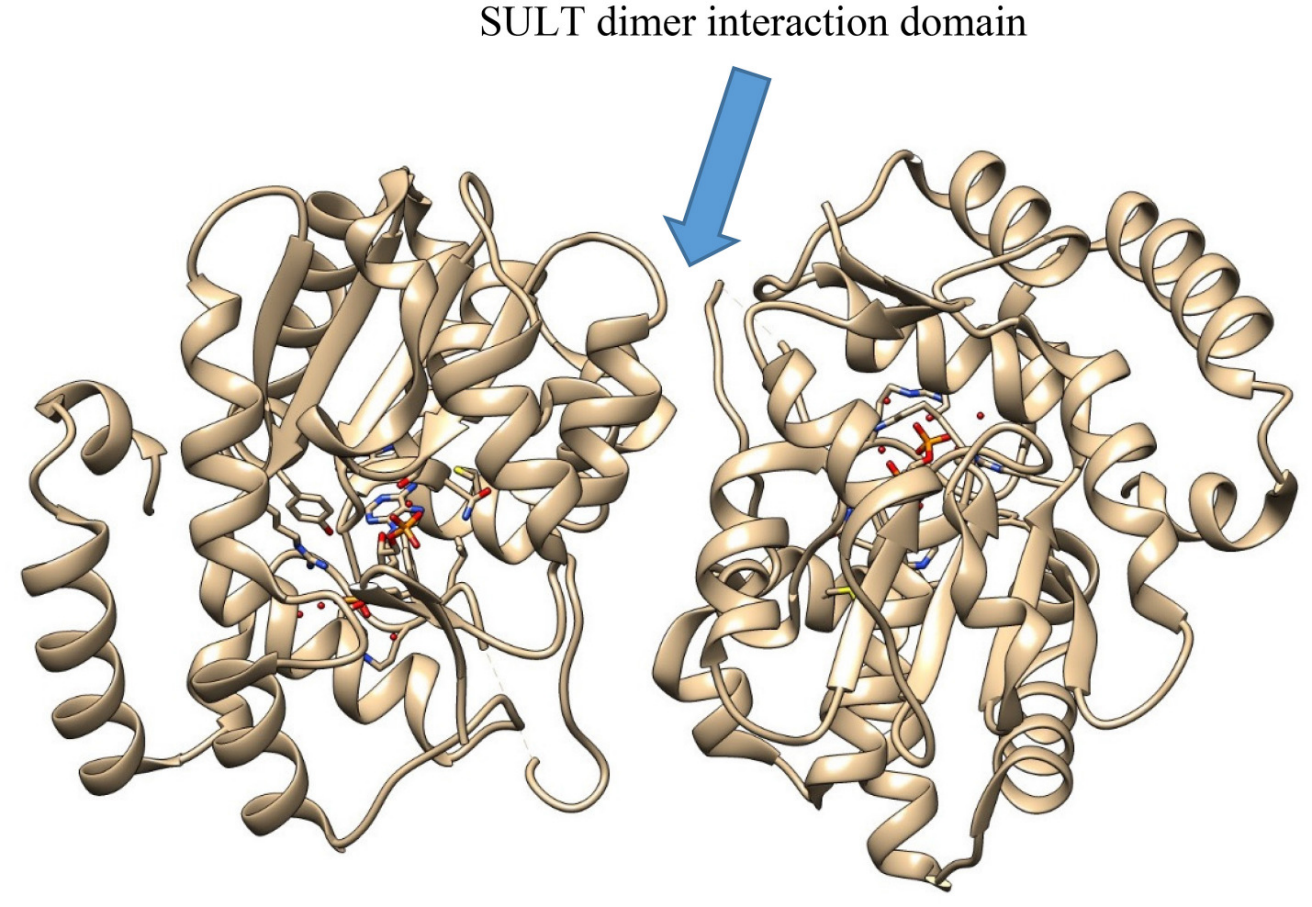
The crystal structure of SULT2A3 (PDB: 1EFH), demonstrating the dimer interaction motif (center).

In SULT enzymes, the dimerization domain (Figure 3) consists of ten residues near the C-terminus of the protein, represented by the consensus sequence KXXXTVXXXE, also known as the KTVE motif (Petrotchenko et al., 2001). In contrast to the interaction interface observed with GST enzymes, this region forms a hydrophilic loop that creates multiple contacts between the two individual monomers. This is reinforced by multiple hydrophobic contacts internal to the KTVE motif, resulting in a “dumbbell”-like structure (Petrotchenko et al., 2001; Yoshinari et al., 2001). While the exact role dimerization plays in catalysis is not totally understood in these enzyme families, it is likely that future X-ray crystal structures will help elucidate it.

Unfortunately, our structural understanding of UGT enzymes is much more limited. While there is ample biophysical evidence to support the existence of UGT dimers (Meech and Mackenzie, 1997; Lewis et al., 2011; Suzuki et al., 2014), the lack of structural data has hampered protein-protein interaction research with this enzyme class. Interestingly, the structural data that does exist for human UGT enzymes, that of a partial UGT2B7 structure, does provide an indication for the presence of homo dimers (Miley et al., 2007; Fujiwara et al., 2016; Audet-Delage et al., 2017). In the asymmetric unit, the C terminus of one monomer packs into the predicted UDPGA binding site of the other monomer (Miley et al., 2007). At first glance, this may seem somewhat counter intuitive, as the UDPGA co-factor is needed for enzymatic activity. However, the UDPGA binding site of the other UGT2B7 monomer in the asymmetric unit is not occluded, indicating that this monomer may be active while the other sub-unit is blocked. As Miley and co-workers demonstrated, removal of the C-terminal residues, in an effort to eliminate blockage of the UDPGA binding site, produced protein samples that were highly unstable and could not be crystallized (Miley et al., 2007). This may suggest that the blockage of the UDPGA co-factor binding site serves as a form of regulatory control of enzymatic activity, although this has not yet been established.

The situation with the MAO enzymes is a bit more complex, with MAO-A being represented as a monomer, while MAO-B is a functional homodimer (Binda et al., 2001, 2011). Comparison studies between the human and rat MAO-A enzymes, with the latter also being a dimer, have suggested that dimerization increases structural stability and may directly influence the kinetic properties of the individual enzymes (Wang and Edmondson, 2007). Others have hypothesized that dimerization may be essential to orient the protein dipole-dipole moment toward the anionic membrane surface in order to promote catalysis (Binda et al., 2011). While human MAO-A crystalized as a monomer, it is not entirely clear if this is an artifact of the crystallization conditions, or has direct functional relevance, as MAO-A is thought to be a dimer in the membrane bound form of the enzyme (Binda et al., 2011). This illustrates a critical point in examining protein-protein interactions with membrane bound proteins: protein-protein interactions are highly dependent on local conditions, particularly the presence/absence of lipid.

Soluble EH (sEH) has also been observed to form dimers upon crystallization (Argiriadi et al., 1999). The sEH dimer structure itself is stabilized by “domain-swapped” or “handshake” architecture, whereby the vestigial and catalytic domains adopt unrelated α/β folds and are connected by a 16-residue, proline-rich linker (Thr-219–Asp-234). A “domain-swapped” architecture occurs when the domain of one monomer is displaced by the same domain of the other monomer in the asymmetric unit thereby forming a stable dimer interface (Gill, 1983). While the vestigial active site does not participate in epoxide hydrolysis, the vestigial domain plays a critical structural role by stabilizing the dimer. More recent studies have confirmed the importance of dimerization for enzymatic activity (Nelson et al., 2013). Moreover, it has been suggested that the dimer interface may make an attractive target for small molecule therapeutics designed against sEH (Nelson et al., 2013; Matsumoto et al., 2014).

## Nuclear magnetic resonance (NMR)

A powerful and complementary technique to X-ray crystallography is protein NMR (Lampe et al., 2008, 2010; Vallurupalli et al., 2008; Raman et al., 2010; Ahuja et al., 2013; Hiruma et al., 2013; Basudhar et al., 2015). In particular, solution NMR can report not only on protein tertiary structure, but also on conformational dynamics which are important to the interaction with protein partners (Lampe et al., 2008, 2010). An additional advantage is that protein NMR can be performed in the presence of lipid bilayer mimetics, such as bicells (Ahuja et al., 2013) or nanodiscs (Kijac et al., 2007; Gluck et al., 2009), allowing the investigator to examine the effect of lipid on protein-protein interactions. Protein NMR has been most widely used to examine interactions between CYP enzymes and their electron transfer partners (Ahuja et al., 2013; Estrada et al., 2013, 2014, 2016). The Scott lab has been a leader in this area, examining the interactions of CYP17A1 with *b*_5_ (Estrada et al., 2013, 2014) and the FMN domain of reductase (Estrada et al., 2016). This system has a particular advantage in that multiple types of interaction can be monitored through titrations involving substrate, CYP, *b*_5_, reductase FMN domain, or any combination thereof. One important result to come out of these studies is that the strength of the CYP17A1- *b*_5_ interaction is dependent on the identity of the substrate, with the CYP17A1- *b*_5_ interaction being stronger when the hydroxylase substrate pregnenolone is present in the CYP17A1 active site than when the lyase substrate 17α-hydroxypregnenolone is bound (Estrada et al., 2013). In these titration experiments, only the *b*_5_ protein was isotopically labeled with ^15^N. When the “reverse” titration experiment was conducted (i.e., where the CYP17A1 molecule is ^15^N labeled and the *b*_5_ protein was isotopically “silent”), the results were similar (Estrada et al., 2014). Additionally, titration of *b*_5_ into the ^15^N-labeled CYP17A1-pregnenolone complex induced a set of conformational substates closely resembling those of CYP17A1-17α-hydroxypregnenolone complex without *b*_5_, suggesting that *b*_5_ may also be able to allosterically induce enzymatically productive conformations in CYP17A1, even in the absence of the lyase substrate (Estrada et al., 2013, 2014). While these data have yet to be replicated with any other drug metabolizing CYP enzymes, it is intriguing to postulate that the results may be extrapolated to *b*_5_ interactions with other CYPs.

As noted above, a particular advantage of using NMR to examine protein-protein interactions is that it can tolerate the presence of lipid. This fact was exploited by Ramamoorthy et al. to determine the structure of full-length, membrane bound *b*_5_ in the presence of CYP2B4 (Ahuja et al., 2013). This was the very first time that a structure of *b*_5_ had been determined in the presence of a phospholipid bilayer. The structure confirmed the electrostatic nature of the CYP-*b*_5_ interaction, revealing a large number of charge-charge interactions between surface residues on CYP2B4 and *b*_5_ enabling complex formation between the two proteins (Ahuja et al., 2013). Interestingly, it also hinted at the importance of hydrophobic interactions between the complex and the phospholipid bilayer. Confirming the result observed by Estrada et al. when examining the CYP17A1- *b*_5_ interaction, Ramamoorthy et al. also observed increased affinity between the CYP and *b*_5_ in the presence of a small molecule substrate or inhibitor. Finally, their data suggested a pathway for electron transfer between *b*_5_ and CYP2B4, mediated through a salt bridge from the heme propionates of *b*_5_ with Arg125 of CYP2B4. More recently, Ramamoorthy et al. have extended their original studies to examine the interactions of the CYP-*b*_5_ complex using phospholipid bilayer nanodiscs (Zhang et al., 2016). The advantage of this versatile phospholipid bilayer memetic is that it permits exquisite control over the composition of lipid, allowing researchers to examine the effects of various lipid components on structure and catalysis. The examples above illustrate the advantages of 2D protein NMR to obtain detailed structural data for protein-protein interactions in the CYP enzyme family.

Almost 20 years ago, Lian pioneered a novel approach combining both X-ray crystallography and NMR to study homo- and hetero-dimeric interactions in the GST A1-1 isoform (Lian, 1998). In the crystal structure of GST A1-1, the hydrophobic C-terminal region of the protein is highly disordered and absent from the structure, as is often the case with dynamic regions of proteins. Lian's group was able to resolve this region of the protein in the 2D HSQC spectrum and identify residues that might be involved in ligand binding and/or protein-protein interactions. Since that time, a number of studies relying on protein NMR have been conducted using GST enzymes (McCallum et al., 1999; Mahajan et al., 2006).

While fewer structural NMR studies have been carried out with the SULT family of enzymes, a recent novel example of NMR, combined with the use of a nitrox spin label positioned in dynamic regions of the enzyme, gave information regarding dimer interaction and the structure of the catechin ligand binding site on the protein (Cook et al., 2016). This was accomplished by replacing each cysteine residue in the protein with an unreactive residue, then selectively reincorporating a cysteine at each position of interest and reacting the protein with a nitroxyl-oxygen spin label (3-maleimido-PROXYL). The label was attached to the protein at six individual cysteine positions in the protein in order to saturate the dimer in a paramagnetic field of sufficient strength to detect its effects on the solution NMR spectrum, without compromising the catalytic integrity of the enzyme. The design allowed the entire surface of the enzyme to be covered in a detectible paramagnetic field and allowed ligands to be positioned within the active site by triangulating their protons from multiple spin labels attached at various positions within the active site (Cook et al., 2016). After obtaining the NMR spectrum for each mutant, the spectrum was overlaid on the previously determined crystal structure and the final structure was obtained using distance-constrained molecular dynamics docking. This approach was advantageous, and preferred over others, in that: (1) it is applicable over a wide range of ligand affinities, (2) does not require much protein, (3) does not require an isotopic label, and (4) has essentially no molecular weight limitations. Moreover, the same strategy may apply to other drug metabolizing enzyme protein-protein complexes that have limiting cysteine residues.

Progress in applying NMR to the study of UGT enzymes has been slower due to the integral attachment to cellular membranes but, as in the case of the CYP enzymes, it is likely that newly emerging technologies, such as nanodiscs and membrane bicells will be useful in defining their interaction with protein partners.

## Fluorescence technologies

The use of both endogenous and exogenous fluorescence labels for conducting protein-protein interaction studies has a long history (Lakowicz, 1999). Indeed, they have been used very effectively in defining the structural determinants and dynamics required for interaction among drug metabolizing enzymes (Nisimoto et al., 1983; Schwarze et al., 1983; Wu and Yang, 1984; Centeno and Gutierrez-Merino, 1992; Davydov et al., 1996, 2001). Most studies to date have preferred to use exogenous fluorophores due to the fact that they typically exhibit much higher quantum yields than the endogenous fluorophores; tryptophan, tyrosine, phenylalanine, and the fluorescent prosthetic groups.

Unarguablely, the greatest use of fluorescence to monitor protein-protein interactions has been with the CYP enzymes. A key early study, suggesting the presence of CYP homodimers in the ER membrane, involved monitoring the fluorescence anisotropy of the substrate diphenylhexatriene as a proxy for membrane rigidity (Gut et al., 1985). Fluorescence anisotropy is the phenomena by which fluorophore containing molecules emit polarized light when the exciting light source is also polarized. The degree of the polarization of the emitted light is proportional to the anisotropy (r), or rotational motion, of the molecule (Lakowicz, 1999). This can be a powerful technique to determine the relative size of molecules or macromolecular complexes, and has been exploited as such in antibody-based assays, among others (Gorovits and Horowitz, 1995; Lim et al., 1995). In a similar fashion, fluorescence depolarization has also been extensively used to monitor CYP-CYP interactions as well (Greinert et al., 1979, 1982; Schwarz et al., 1993).

Other aspects of fluorescence have also been exploited in studying CYP protein-protein interactions. The technique of fluorescence resonance energy transfer (FRET), whereby an excited donor molecule is able to quantitatively transfer energy to an acceptor molecule that then emits in another region of the spectrum, has been used successfully in several studies of protein-protein interactions among drug metabolizing enzymes (Nisimoto et al., 1983; Schwarze et al., 1983; Davydov et al., 1996, 2000, 2010, 2013a, 2015; Szczesna-Skorupa et al., 2003; Praporski et al., 2009). A singular advantage of this fluorescence technique is that it allows for an indirect measurement of distance between the two fluorophores due to the phenomena of Förster energy transfer (Lakowicz, 1999). In an elegant FRET study conducted in 2003, Kemper et al. were able to observe a direct physical interaction between individual CYPs in a live cell membrane utilizing CYP2E1 and CYP2C2 which were labeled with donor and acceptor fluorophores in cells transfected with murine CYP cDNA (Szczesna-Skorupa et al., 2003). They discovered that, while FRET occurred between individual CYP2C2 molecules in a membrane, it could not be detected between CYP2E1 monomers, representing a homomeric self-association with CYP2C2, but not CYP2E1. Later work confirmed the existence of the CYP2C2 dimers in murine hepatocyte endoplasmic reticulum membranes, demonstrating the potential *in vivo* relevance of these types of protein-protein interactions (Li et al., 2011).

Sligar's group at the University of Illinois was an early adopter of fluorescence technologies to monitor CYP interactions with effector proteins, such as *b*_5_ (Stayton et al., 1988). By selectively replacing threonine residues with cysteine in *b*_5_, they were able to site-specifically introduce the sulfhydryl selective fluorescent reagent, acrylodan, at different positions in the protein. Acrylodan generally reacts with accessible thiol groups more slowly than maleimides or iodoacetamides, but tends to form highly stable thioether bonds (Weber and Farris, 1979). Additionally, in the excited state there is a substantial charge separation between the amino and carbonyl groups, which produces large spectral shifts in a hydrophobic environment, making it an ideal spectral probe for monitoring protein-protein interactions. When the labeled *b*_5_ protein was allowed to interact with CYP P-450cam, a model bacterial isoform, a significant fluorescence enhancement and blue shift was observed, indicating the fluorophore's transition to a more hydrophobic binding site and an increased binding free energy for the two proteins.

More recently, Usanov et al. expanded on the Sligar lab's early work by using a chimeric *b*_5_-GFP to examine protein-protein interactions between *b*_5_ and CYP3A4 (Yantsevich et al., 2009). Utilizing this chimeric construct, the authors were able to determine binding affinities between various *b*_5_ and CYP3A4 complexes. An interesting result from this report was that the hydrophobic domain of *b*_5_ was observed to participate both in hemeprotein interaction and electron transfer directly from *b*_5_ to the CYP enzyme. Simonov et al. used a CYP17A1 with eCFP fused to the C-terminus and eYFP fused to the N-terminus of *b*_5_ to examine CYP17A1- *b*_5_ protein-protein interactions using FRET (Simonov et al., 2015). They combined this technique with structural modeling and molecular dynamics simulations to show that *b*_5_ interacts directly with CYP17A1 in the ER membrane to stimulate the lyase reaction.

Fluorescence tools have also proven useful to examine CYP-CPR interactions. A unique approach by Davydov et al. to study the effect of substrate binding on CYP protein-protein interactions involved substituting the ferric-protoprophyrin IX heme with an aluminum-protoporphyrin IX (Nisimoto et al., 1983). While previous studies had used zinc substituted protoporphyrins, the replacement of the iron atom with aluminum resulted in an isosteric displacement, since both transition metals have approximately the same interatomic radius. Therefore, the aluminum substitution was unlikely to have a major effect on the overall structure of the active site. Additionally, while the aluminum atom is not able to donate or accept electrons, it imparts a high degree of fluorescence to the protoprophyrin, thereby introducing an internal probe which allowed the authors to monitor the interactions between CYP P450 BM-3 and its substrates and protein partners using fluorescence energy transfer (Davydov et al., 2013b). The CYP P450 BM-3 retained its ability to interact with the reductase domain despite the substitution in the protoprophyrin and the newly imparted fluorescence from the aluminum-protoporphyrin IX allowed the authors to determine affinity between the CYP domain and the reductase domain.

Fluorescence studies have also been useful in the study of the human UGT enzymes (Operana and Tukey, 2007; Fujiwara et al., 2016; Yuan et al., 2016). Turkey et al. used FRET to examine UGT1A oligomerization *in vivo* (Operana and Tukey, 2007). Using this technique, they were able to show that UGT1A1, 1A3, 1A4, 1A6, 1A7, 1A8, 1A9, and UGT1A10 all form homodimers in live cells. Additionally, the authors confirmed heterodimer interactions between UGT1A1 and the UGT1A3, 1A4, 1A6, 1A7, 1A8, 1A9, and UGT1A10 isoforms. More recently, Yuan et al. used FRET to demonstrate that UGT2B7 was able to form homo oligomers with both wild-type and mutant forms of the enzyme, which had the possibility of affecting zidovudine glucuronidation (Yuan et al., 2015). Yuan further went on to demonstrate that the UGT isoforms UGT1A1, 1A9, and 2B7 were all able to form heterodimeric complexes, expanding on their previous work that examined homodimerization in this enzyme class (Yuan et al., 2016).

Beckman et al took a unique approach to studying intersubunit communication in dimeric complexes of SULT enzymes, by examining fluorescence quenching of the enzyme's intrinsic fluorophores upon binding of the inhibitors, 2,6-dichloro-4-nitrophenol (DCNP) and pentachlorophenol (PCP; Beckmann et al., 1998). Using this technique, the authors were able to determine that binding of the co-factor 3′-phosphoadenosine-5′-phosphosulfate (PAPS) facilitated positive cooperative binding between the individual subunits. This illustrates the use of a powerful fluorescence technique that does not rely on introduction of an extrinsic fluorophore to obtain information on protein-protein interactions, allowing for an examination of protein-protein interactions in the native state of the enzyme.

Another seminal FRET study, this time involving GST P1-1, demonstrated that the C-terminus of c-Jun N-terminal kinase could interact directly with this GST enzyme (Wang et al., 2001). This was an early study that identified a role for GST P1-1 in regulating certain kinase pathways. The Atkins group has used both intrinsic fluorescence of proteins (Wang et al., 1993; Dietze et al., 1996) and of ligands (Lu and Atkins, 2004) to examine the interaction of GST dimers and their substrates.

## Photo-affinity labels and crosslinking studies

Photoaffinity labels and chemical crosslinking agents have long been recognized as useful tools to understand protein-protein interactions. Photoaffinity labels are particularly useful if they are also enzyme substrates, as they are typically localized at the CYP active site until activated with irradiation (Wen et al., 2005, 2006). Wen and Lampe utilized the photoaffinity label lapachenole in combination with cysteine-scanning mutagenesis to identify specific residues of CYP3A4 that interact with CPR, including cysteine 98, using LC-MS (Wen et al., 2006). Similarly, Sulc et al. employed the photoaffinity ligand 3-azidiamantane to label CYP2B1 (Hodek and Smrcek, 1999) and CYP2B4 (Sulc et al., 2008). While, in this case, the diamantoid probe did not function specifically as a probe of protein-protein interactions, it has the potential to report on specific conformational changes in the CYP enzyme that may accompany interaction with protein partners.

In general, crosslinking agents are more versatile and have been used more extensively in assessing protein-protein interactions. In particular, LC-MS combined with crosslinking, can be powerful in detecting specific interactions between two proteins that are known to interact. However, the non-specificity of most chemical crosslinking agents make the large datasets generated from such experiments difficult to analyze. Gao et al. developed a software tool called Pro-CrossLink to address this specific issue (Gao et al., 2006a). Using this tool, combined with a site-directed mutagenesis approach and the standard crosslinking agent, 1-ethyl-3-[3-dimethylaminopropyl]carbodiimide hydrochloride (EDC), they were able to identify relevant protein-protein interactions that occurred between CYP2E1 and *b*_5_ (Gao et al., 2006b). Their strategy relied on an ^18^O-labeling method that incorporated twice as many ^18^O atoms in cross-linked peptides as non-cross-linked peptides when proteolysis was conducted in ^18^O-labeled water. Subsequent tandem mass spectrometric (MS/MS) analysis of the selected cross-linked peptide candidates led to the identification of two intermolecular cross-links, one at K428-CYP2E1 to D53-b5 and K434-CYP2E1 to the E56 residue of *b*_5_. These results provided some of the first direct biophysical evidence for the interacting orientations of a microsomal CYP and *b*_5_.

More recently, investigators have used chemical crosslinkers to identify CYP interaction with other, non-canonical proteins. Using the amine-reactive EGS crosslinker, Losel et al. was able to demonstrate that PGRMC1, a known CYP modulator, interacts with a yet-to-be-identified 52 kDa protein, presumably a CYP enzyme, in pig liver microsomes (Losel et al., 2008). Additionally, substantial progress has been made examining the homodimeric interactions of other CYPs, such as CYP3A4 (Davydov et al., 2015) and others (Reed et al., 2010) using crosslinking technology.

## SPR and surface immobilization

Surface Plasmon Resonance (SPR) and related surface biolayer technologies have been mainstay technologies for examining protein-protein interactions in a variety of systems for many years (Cooper, 2002). These technologies rely on immobilization of the target, or “receptor,” protein onto the solid surface of a chip (usually Au), then a (potential) protein partner, the “ligand,” is allowed to flow over the surface of the chip and, if binding occurs, this results in a cascading plasmon wave in the gold layer that causes a detectable change in the refractive index of an incident light source (usually a laser) that is directly proportional to the binding interaction (Cooper, 2002). Early studies with CYP enzymes demonstrated the sensitivity of this technology in monitoring ligand binding directly to CYP enzymes, paving the way for more sophisticated protein-protein interaction studies (Pearson et al., 2006). More recently, this technology has been applied to examining CYP interactions with electron transfer partners (Ivanov et al., 1997, 1999, 2001; Kuznetsov et al., 2004; Archakov and Ivanov, 2011; Yablokov et al., 2017).

Yablokov et al. used a modified construct of *b*_5_ covalently attached through an NHS linkage to a gold SPR chip to directly monitor the interactions between *b*_5_ and various human CYP isoforms, including both CYP's whose activity was allosterically regulated by *b*_5_ and those whose activity was unchanged in the presence of *b*_5_ (Yablokov et al., 2017). Interestingly, they determined that the CYP-*b*_5_ interactions fell into two classes: those that were enthalpy driven and those that were entropy driven. The CYP-*b*_5_ interactions that were enthalpy driven tended to belong to CYPs that were allosterically regulated by *b*_5_ (i.e., whose activity could be modulated by *b*_5_), whereas the CYP-*b*_5_ interactions that were entropy driven tended to represent the CYP's whose activity was unaffected by *b*_5_. The authors attributed the differences in these effects to positive ΔH values corresponding to displacement of the solvation shells of proteins upon clustering (Yablokov et al., 2017).

Gannett and his team applied SPR to examine CYP-CYP interactions between CYP2C9, CYP3A4, CYP2D6, and other isoforms (Bostick et al., 2016). Somewhat surprisingly, they found that the highest affinity complex was formed between CYP2C9 and CYP2D6, a heterodimeric complex, with the affinity between CYP2C9 and CPR being lower than that of heterodimers and CYP2C9 homodimers. Additionally, they observed that the affinities of specific complexes were highly dependent on the order of addition of the individual proteins involved.

Previously, in a clever application combining two technologies, Shimada and Guengerich used immobilized CYP proteins, coupled with an enzyme-linked affinity approach, to measure relative affinities between the CYP-CPR complexes and CYP-*b*_5_ complexes (Shimada et al., 2005). Individual CYP proteins were purified and bound to separate wells of a polystyrene plate, after which the biotinylated partner enzymes were added. Finally, a streptavidin-peroxidase complex was added to each well and protein-protein interaction was monitored by measuring peroxidase activity of the bound biotinylated proteins. This allowed for an indirect calculation of K_*d*_'s for the individual CYPs to CPR and *b*_5_.

The ever-expanding and versatile nature of the SPR platform(s) indicate that this technology will continue to be useful for monitoring protein-protein interactions in the immediate future and beyond.

## Emerging biophysical technologies to examine protein-protein interactions and future perspective

Today is an exciting time to study protein-protein interactions in drug metabolizing enzymes. A number of new technologies are beginning to emerge that have the power to revolutionize our understanding of how protein-protein interactions modulate the activity of these important proteins. Quartz crystal microbalance studies have helped define the protein-protein interactions between CYP17A1 and *b*_5_, in part explaining enzymatic product distribution ratios (Simonov et al., 2015). Additionally, these types of studies have also been useful in identifying sites of protein-protein interaction in CYP19 (aromatase; Martin et al., 2015). Along with quartz crystal microbalance studies, conductometric monitoring is another emerging technology that has the potential to provide information on protein-protein interaction in drug metabolizing enzymes (Spera et al., 2013).

In conclusion, the future use of traditional and emerging biophysical technologies to monitor protein-protein interactions is likely to provide us with further insight to the function and regulation of these enzymes critically important for drug metabolism and disposition.

## Author contributions

The author confirms being the sole contributor of this work and approved it for publication.

### Conflict of interest statement

The author declares that the research was conducted in the absence of any commercial or financial relationships that could be construed as a potential conflict of interest.

## Abbreviations

CYP: cytochrome P450
CPR: cytochrome P450 reductase
b5: cytochrome b5
SULT: sulfotransferase
GST: glutathione-S-transferase
HLM: human liver microsomes
HO: heme oxygenase
UGT: uridine 5′-diphospho-glucuronosyltransferase
UDPGA: uridine 5′-diphospho-glucuronic acid
MAO: monoamine oxidase
sEH: soluble epoxide hydrolase
2D NMR: two dimensional nuclear magnetic resonance
Pdx: putidaredoxin
SLF: separated local field
2D PELF: two dimensional proton evolved local field
INEPT: insensitive nuclei enhanced by polarization transfer
DREPT: dipolar enhanced polarization transfer
FRET: fluorescence resonance energy transfer
NHS: *N*-Hydroxysuccinimide
ER: endoplasmic reticulum
SER: smooth endoplasmic reticulum
PGRMC1: progesterone receptor membrane component 1
PAPS: 3′-phosphoadenosine-5′-phosphosulfate.

## References

[B1] Abdalla A. M. Bruns C. M. Tainer J. A. Mannervik B. Stenberg G. (2002). Design of a monomeric human glutathione transferase GSTP1, a structurally stable but catalytically inactive protein. Protein Eng. 15, 827–834. 10.1093/protein/15.10.827 12468717

[B2] Ahuja S. Jahr N. Im S. C. Vivekanandan S. Popovych N. Le Clair S. V.. (2013). A model of the membrane-bound cytochrome b5-cytochrome P450 complex from NMR and mutagenesis data. J. Biol. Chem. 288, 22080–22095. 10.1074/jbc.M112.448225 23709268 PMC3724662

[B3] Archakov A. I. Ivanov Y. D. (2011). Application of AFM and optical biosensor for investigation of complexes formed in P450-containing monooxygenase systems. Biochim. Biophys. Acta 1814, 102–110. 10.1016/j.bbapap.2010.08.013 20832504

[B4] Argiriadi M. A. Morisseau C. Hammock B. D. Christianson D. W. (1999). Detoxification of environmental mutagens and carcinogens: structure, mechanism, and evolution of liver epoxide hydrolase. Proc. Natl. Acad. Sci. U.S.A. 96, 10637–10642. 10.1073/pnas.96.19.10637 10485878 PMC17935

[B5] Audet-Delage Y. Rouleau M. Rouleau M. Roberge J. Miard S. Picard F.. (2017). Cross-Talk between alternatively spliced UGT1A Isoforms and Colon Cancer Cell Metabolism. Mol. Pharmacol. 91, 167–177. 10.1124/mol.116.106161 28049773

[B6] Balogh L. M. Le Trong I. Kripps K. A. Tars K. Stenkamp R. E. Mannervik B.. (2009). Structural analysis of a glutathione transferase A1-1 mutant tailored for high catalytic efficiency with toxic alkenals. Biochemistry 48, 7698–7704. 10.1021/bi900895b 19618965 PMC2753285

[B7] Basudhar D. Madrona Y. Kandel S. Lampe J. N. Nishida C. R. de Montellano P. R. (2015). Analysis of cytochrome P450 CYP119 ligand-dependent conformational dynamics by two-dimensional NMR and X-ray crystallography. J. Biol. Chem. 290, 10000–10017. 10.1074/jbc.M114.627935 25670859 PMC4400317

[B8] Beckmann J. D. Henry T. Ulphani J. Lee P. (1998). Cooperative ligand binding by bovine phenol sulfotransferase. Chem. Biol. Interact. 109, 93–105. 10.1016/S0009-2797(97)00123-3 9566736

[B9] Binda C. Angelini R. Federico R. Ascenzi P. Mattevi A. (2001). Structural bases for inhibitor binding and catalysis in polyamine oxidase. Biochemistry 40, 2766–2776. 10.1021/bi002751j 11258887

[B10] Binda C. Mattevi A. Edmondson D. E. (2011). Structural properties of human monoamine oxidases A and B. Int. Rev. Neurobiol. 100, 1–11. 10.1016/B978-0-12-386467-3.00001-7 21971000

[B11] Bostick C. D. Hickey K. M. Wollenberg L. A. Flora D. R. Tracy T. S. Gannett P. M. (2016). Immobilized Cytochrome P450 for Monitoring of P450-P450 interactions and metabolism. Drug Metab. Dispos. 44, 741–749. 10.1124/dmd.115.067637 26961240 PMC4851305

[B12] Centeno F. Gutierrez-Merino C. (1992). Location of functional centers in the microsomal cytochrome P450 system. Biochemistry 31, 8473–8481. 10.1021/bi00151a013 1390631

[B13] Chenge J. T. Duyet L. V. Swami S. McLean K. J. Kavanagh M. E. Coyne A. G.. (2017). Structural characterization and Ligand/Inhibitor identification provide functional insights into the *Mycobacterium tuberculosis* Cytochrome P450 CYP126A1. J. Biol. Chem. 292, 1310–1329. 10.1074/jbc.M116.748822 27932461 PMC5270475

[B14] Cook I. Wang T. Girvin M. Leyh T. S. (2016). The structure of the catechin-binding site of human sulfotransferase 1A1. Proc. Natl. Acad. Sci. U.S.A. 113, 14312–14317. 10.1073/pnas.1613913113 27911811 PMC5167148

[B15] Cooper M. A. (2002). Optical biosensors in drug discovery. Nat. Rev. Drug Discov. 1, 515–528. 10.1038/nrd838 12120258

[B16] Cupp-Vickery J. Anderson R. Hatziris Z. (2000). Crystal structures of ligand complexes of P450_*eryF*_ exhibiting homotropic cooperativity. Proc. Natl. Acad. Sci. U.S.A. 97, 3050–3055. 10.1073/pnas.97.7.3050 10716705 PMC16190

[B17] Davydov D. R. (2011). Microsomal monooxygenase as a multienzyme system: the role of P450-P450 interactions. Expert Opin. Drug Metab. Toxicol. 7, 543–558. 10.1517/17425255.2011.562194 21395496 PMC3079778

[B18] Davydov D. R. (2016). Molecular organization of the microsomal oxidative system: a new connotation for an old term. Biochem. Suppl. Ser. B 10, 10–21. 10.1134/S1990750816010042 25978385

[B19] Davydov D. R. Davydova N. Y. Sineva E. V. Halpert J. R. (2015). Interactions among cytochromes P450 in microsomal membranes: oligomerization of cytochromes P450 3A4, 3A5, and 2E1 and its functional consequences. J. Biol. Chem. 290, 3850–3864. 10.1074/jbc.M114.615443 25533469 PMC4319048

[B20] Davydov D. R. Davydova N. Y. Sineva E. V. Kufareva I. Halpert J. R. (2013a). Pivotal role of P450-P450 interactions in CYP3A4 allostery: the case of alpha-naphthoflavone. Biochem. J. 453, 219–230. 10.1042/BJ20130398 23651100 PMC3893034

[B21] Davydov D. R. Kariakin A. A. Petushkova N. A. Peterson J. A. (2000). Association of cytochromes P450 with their reductases: opposite sign of the electrostatic interactions in P450BM-3 as compared with the microsomal 2B4 system. Biochemistry 39, 6489–6497. 10.1021/bi992936u 10828964

[B22] Davydov D. R. Knyushko T. V. Kanaeva I. P. Koen Y. M. Samenkova N. F. Archakov A. I.. (1996). Interactions of cytochrome P450 2B4 with NADPH-cytochrome P450 reductase studied by fluorescent probe. Biochimie 78, 734–743. 10.1016/S0300-9084(97)82531-X 9010602

[B23] Davydov D. R. Petushkova N. A. Bobrovnikova E. V. Knyushko T. V. Dansette P. (2001). Association of cytochromes P450 1A2 and 2B4: are the interactions between different P450 species involved in the control of the monooxygenase activity and coupling? Adv. Exp. Med. Biol. 500, 335–338. 10.1007/978-1-4615-0667-6_53 11764964

[B24] Davydov D. R. Ponomarev G. V. Bobrovnikova-Marjon E. Haines D. C. Peterson J. A. (2013b). Aluminum-substituted heme domain of P450BM-3 (BMP): introducing a heme-derived fluorescent probe for studies of substrate binding and protein-protein interactions in cytochromes P450. Biotechnol. Appl. Biochem. 60, 41–51. 10.1002/bab.1085 23586991

[B25] Davydov D. R. Sineva E. V. Sistla S. Davydova N. Y. Frank D. J. Sligar S. G.. (2010). Electron transfer in the complex of membrane-bound human cytochrome P450 3A4 with the flavin domain of P450BM-3: the effect of oligomerization of the heme protein and intermittent modulation of the spin equilibrium. Biochim. Biophys. Acta 1797, 378–390. 10.1016/j.bbabio.2009.12.008 20026040 PMC2819549

[B26] Deng B. Parthasarathy S. Wang W. Gibney B. R. Battaile K. P. Lovell S.. (2010). Study of the individual cytochrome b5 and cytochrome b5 reductase domains of Ncb5or reveals a unique heme pocket and a possible role of the CS domain. J. Biol. Chem. 285, 30181–30191. 10.1074/jbc.M110.120329 20630863 PMC2943328

[B27] Dietze E. C. Wang R. W. Lu A. Y. Atkins W. M. (1996). Ligand effects on the fluorescence properties of tyrosine-9 in alpha 1-1 glutathione S-transferase. Biochemistry 35, 6745–6753. 10.1021/bi9530346 8639625

[B28] Estrada D. F. Laurence J. S. Scott E. E. (2013). Substrate-modulated cytochrome P450 17A1 and cytochrome b5 interactions revealed by NMR. J. Biol. Chem. 288, 17008–17018. 10.1074/jbc.M113.468926 23620596 PMC3675632

[B29] Estrada D. F. Laurence J. S. Scott E. E. (2016). Cytochrome P450 17A1 Interactions with the FMN Domain of Its Reductase as Characterized by NMR. J. Biol. Chem. 291, 3990–4003. 10.1074/jbc.M115.677294 26719338 PMC4759177

[B30] Estrada D. F. Skinner A. L. Laurence J. S. Scott E. E. (2014). Human cytochrome P450 17A1 conformational selection: modulation by ligand and cytochrome b5. J. Biol. Chem. 289, 14310–14320. 10.1074/jbc.M114.560144 24671419 PMC4022897

[B31] Evers R. Dallas S. Dickmann L. J. Fahmi O. A. Kenny J. R. Kraynov E.. (2013). Critical review of preclinical approaches to investigate cytochrome p450-mediated therapeutic protein drug-drug interactions and recommendations for best practices: a white paper. Drug Metab. Dispos. 41, 1598–1609. 10.1124/dmd.113.052225 23792813

[B32] Fujiwara R. Yokoi T. Nakajima M. (2016). Structure and Protein-Protein Interactions of Human UDP-Glucuronosyltransferases. Front. Pharmacol. 7:388. 10.3389/fphar.2016.00388 27822186 PMC5075577

[B33] Gao Q. Doneanu C. E. Shaffer S. A. Adman E. T. Goodlett D. R. Nelson S. D. (2006b). Identification of the interactions between cytochrome P450 2E1 and cytochrome b5 by mass spectrometry and site-directed mutagenesis. J. Biol. Chem. 281, 20404–20417. 10.1074/jbc.M601785200 16679316

[B34] Gao Q. Xue S. Doneanu C. E. Shaffer S. A. Goodlett D. R. Nelson S. D. (2006a). Pro-CrossLink. Software tool for protein cross-linking and mass spectrometry. Anal. Chem. 78, 2145–2149. 10.1021/ac051339c 16579592

[B35] Gill S. S. (1983). Purification of mouse liver cytosolic epoxide hydrolase. Biochem. Biophys. Res. Commun. 112, 763–769. 10.1016/0006-291X(83)91527-9 6847673

[B36] Gluck J. M. Wittlich M. Feuerstein S. Hoffmann S. Willbold D. Koenig B. W. (2009). Integral membrane proteins in nanodiscs can be studied by solution NMR spectroscopy. J. Am. Chem. Soc. 131, 12060–12061. 10.1021/ja904897p 19663495

[B37] Gorovits B. M. Horowitz P. M. (1995). The molecular chaperonin cpn60 displays local flexibility that is reduced after binding with an unfolded protein. J. Biol. Chem. 270, 13057–13062. 10.1074/jbc.270.22.13057 7768899

[B38] Grahn E. Novotny M. Jakobsson E. Gustafsson A. Grehn L. Olin B.. (2006). New crystal structures of human glutathione transferase A1-1 shed light on glutathione binding and the conformation of the C-terminal helix. Acta Cryst. 62(Pt. 2), 197–207. 10.1107/S0907444905039296 16421451

[B39] Greinert R. Finch S. A. Stier A. (1982). Cytochrome P-450 rotamers control mixed-function oxygenation in reconstituted membranes. Rotational diffusion studied by delayed fluorescence depolarization. Xenobiotica 12, 717–726. 10.3109/00498258209038946 7168192

[B40] Greinert R. Staerk H. Stier A. Weller A. (1979). E-type delayed fluorescence depolarization, technique to probe rotational motion in the microsecond range. J. Biochem. Biophys. Methods 1, 77–83. 10.1016/0165-022X(79)90014-9 95189

[B41] Guengerich F. P. (2006). Cytochrome P450s and other enzymes in drug metabolism and toxicity. AAPS J. 8, E101–E111. 10.1208/aapsj080112 16584116 PMC2751428

[B42] Gut J. Kawato S. Cherry R. J. Winterhalter K. H. Richter C. (1985). Lipid peroxidation decreases the rotational mobility of cytochrome P-450 in rat liver microsomes. Biochim. Biophys. Acta 817, 217–228. 10.1016/0005-2736(85)90023-9 3925992

[B43] Hall D. A. Vander Kooi C. W. Stasik C. N. Stevens S. Y. Zuiderweg E. R. Matthews R. G. (2001). Mapping the interactions between flavodoxin and its physiological partners flavodoxin reductase and cobalamin-dependent methionine synthase. Proc. Natl. Acad. Sci. U.S.A. 98, 9521–9526. 10.1073/pnas.171168898 11493691 PMC55485

[B44] Hamdane D. Xia C. Im S. C. Zhang H. Kim J. J. Waskell L. (2009). Structure and function of an NADPH-cytochrome P450 oxidoreductase in an open conformation capable of reducing cytochrome P450. J. Biol. Chem. 284, 11374–11384. 10.1074/jbc.M807868200 19171935 PMC2670143

[B45] He Y. A. Gajiwala K. S. Wu M. Parge H. Burke B. Lee C. A. (Eds.). (2006). The crystal structure of human CYP3A4 in complex with testosterone. [Abstracts]. in 16th Int Smpos Microsomal Drug Oxidations (MDO 2006). Budapest, Hungary: Diamond Congress, Ltd. (Budapest).

[B46] Henderson C. J. McLaughlin L. A. Wolf C. R. (2013). Evidence that cytochrome b5 and cytochrome b5 reductase can act as sole electron donors to the hepatic cytochrome P450 system. Mol. Pharmacol. 83, 1209–1217. 10.1124/mol.112.084616 23530090

[B47] Henzler-Wildman K. Kern D. (2007). Dynamic personalities of proteins. Nature 450, 964–972. 10.1038/nature06522 18075575

[B48] Hiruma Y. Hass M. A. Kikui Y. Liu W. M. Olmez B. Skinner S. P.. (2013). The structure of the cytochrome p450cam-putidaredoxin complex determined by paramagnetic NMR spectroscopy and crystallography. J. Mol. Biol. 425, 4353–4365. 10.1016/j.jmb.2013.07.006 23856620

[B49] Hodek P. Smrcek S. (1999). Evaluation of 3-azidiamantane as photoaffinity probe of cytochrome P450. Gen. Physiol. Biophys. 18, 181–198. 10517292

[B50] Ivanov Y. D. Kanaeva I. P. Eldarov M. A. Sklyabin K. G. Lehnerer M. Schulze J.. (1997). An optical biosensor study of the interaction parameters and role of hydrophobic tails of cytochrome P450 2B4, b5 and NADPH-flavoprotein in complex formation. Biochem. Mol. Biol. Int. 42, 731–737. 10.1080/15216549700203161 19856290

[B51] Ivanov Y. D. Kanaeva I. P. Karuzina I. I. Usanov S. A. Hui Bon Hoa G. Sligar S. G.. (2001). Revelation of ternary complexes between redox partners in cytochrome P450-containing monooxygenase systems by the optical biosensor method. J. Inorg. Biochem. 87, 175–184. 10.1016/S0162-0134(01)00332-4 11744054

[B52] Ivanov Y. D. Kanaeva I. P. Kuznetsov V. Y. Lehnerer M. Schulze J. Hlavica P.. (1999). The optical biosensor studies on the role of hydrophobic tails of NADPH-cytochrome P450 reductase and cytochromes P450 2B4 and b5 upon productive complex formation within a monomeric reconstituted system. Arch. Biochem. Biophys. 362, 87–93. 10.1006/abbi.1998.0981 9917332

[B53] Kakuta Y. Pedersen L. G. Carter C. W. Negishi M. Pedersen L. C. (1997). Crystal structure of estrogen sulphotransferase. Nat. Struct. Biol. 4, 904–908. 10.1038/nsb1197-904 9360604

[B54] Kandel S. E. Lampe J. N. (2014). Role of protein-protein interactions in cytochrome P450-mediated drug metabolism and toxicity. Chem. Res. Toxicol. 27, 1474–1486. 10.1021/tx500203s 25133307 PMC4164225

[B55] Kijac A. Z. Li Y. Sligar S. G. Rienstra C. M. (2007). Magic-angle spinning solid-state NMR spectroscopy of nanodisc-embedded human CYP3A4. Biochemistry 46, 13696–13703. 10.1021/bi701411g 17985934 PMC2571072

[B56] Kuznetsov V. Y. Ivanov Y. D. Archakov A. I. (2004). Atomic force microscopy revelation of molecular complexes in the multiprotein cytochrome P450 2B4-containing system. Proteomics 4, 2390–2396. 10.1002/pmic.200300751 15274134

[B57] Lakowicz J. R. (1999). Principles of Fluorescence Spectroscopy. 2nd. ed. New York, NY: Kluwer Academic/Plenum Publishers.

[B58] Lampe J. N. Brandman R. Sivaramakrishnan S. de Montellano P. R. (2010). Two-dimensional NMR and all-atom molecular dynamics of cytochrome P450 CYP119 reveal hidden conformational substates. J. Biol. Chem. 285, 9594–9603. 10.1074/jbc.M109.087593 20097757 PMC2843209

[B59] Lampe J. N. Floor S. N. Gross J. D. Nishida C. R. Jiang Y. Trnka M. J.. (2008). Ligand-induced conformational heterogeneity of cytochrome P450 CYP119 identified by 2D NMR spectroscopy with the unnatural amino acid (13)C-p-methoxyphenylalanine. J. Am. Chem. Soc. 130, 16168–16169. 10.1021/ja8071463 18998650 PMC2645923

[B60] Lewis B. C. Mackenzie P. I. Miners J. O. (2011). Homodimerization of UDP-glucuronosyltransferase 2B7 (UGT2B7) and identification of a putative dimerization domain by protein homology modeling. Biochem. Pharmacol. 82, 2016–2023. 10.1016/j.bcp.2011.09.007 21930117

[B61] Li B. Yau P. Kemper B. (2011). Identification of cytochrome P450 2C2 protein complexes in mouse liver. Proteomics 11, 3359–3368. 10.1002/pmic.201100001 21751364 PMC3319039

[B62] Lian L. Y. (1998). NMR structural studies of glutathione S-transferase. Cell. Mol. Life Sci. 54, 359–362. 10.1007/s000180050164 9614973 PMC11147186

[B63] Lim K. Jameson D. M. Gentry C. A. Herron J. N. (1995). Molecular dynamics of the anti-fluorescein 4-4-20 antigen-binding fragment. 2. Time-resolved fluorescence spectroscopy. Biochemistry 34, 6975–6984. 10.1021/bi00021a009 7766607

[B64] Losel R. M. Besong D. Peluso J. J. Wehling M. (2008). Progesterone receptor membrane component 1–many tasks for a versatile protein. Steroids 73, 929–934. 10.1016/j.steroids.2007.12.017 18249431

[B65] Lu W. D. Atkins W. M. (2004). A novel antioxidant role for ligandin behavior of glutathione S-transferases: attenuation of the photodynamic effects of hypericin. Biochemistry 43, 12761–12769. 10.1021/bi049217m 15461448

[B66] Mahajan S. S. Hou L. Doneanu C. Paranji R. Maeda D. Zebala J.. (2006). Optimization of bivalent glutathione S-transferase inhibitors by combinatorial linker design. J. Am. Chem. Soc. 128, 8615–8625. 10.1021/ja061766n 16802828

[B67] Martin L. L. Holien J. K. Mizrachi D. Corbin C. J. Conley A. J. Parker M. W.. (2015). Evolutionary comparisons predict that dimerization of human cytochrome P450 aromatase increases its enzymatic activity and efficiency. J. Steroid Biochem. Mol. Biol. 154, 294–301. 10.1016/j.jsbmb.2015.09.006 26361012

[B68] Matsumoto N. Suzuki E. Ishikawa M. Shirafuji T. Hasumi K. (2014). Soluble Epoxide Hydrolase as an Anti-inflammatory Target of the Thrombolytic Stroke Drug SMTP-7. J. Biol. Chem. 289, 35826–35838. 10.1074/jbc.M114.588087 25361765 PMC4276851

[B69] McCallum S. A. Hitchens T. K. Rule G. S. (1999). Solution structure of the carboxyl terminus of a human class Mu glutathione S-transferase: NMR assignment strategies in large proteins. J. Mol. Biol. 285, 2119–2132. 10.1006/jmbi.1998.2428 9925789

[B70] Meech R. Mackenzie P. I. (1997). UDP-glucuronosyltransferase, the role of the amino terminus in dimerization. J. Biol. Chem. 272, 26913–26917. 10.1074/jbc.272.43.26913 9341125

[B71] Miley M. J. Zielinska A. K. Keenan J. E. Bratton S. M. Radominska-Pandya A. Redinbo M. R. (2007). Crystal structure of the cofactor-binding domain of the human phase II drug-metabolism enzyme UDP-glucuronosyltransferase 2B7. J. Mol. Biol. 369, 498–511. 10.1016/j.jmb.2007.03.066 17442341 PMC1976284

[B72] Murakami Y. Tripathi L. P. Prathipati P. Mizuguchi K. (2017). Network analysis and *in silico* prediction of protein-protein interactions with applications in drug discovery. Curr. Opin. Struct. Biol. 44, 134–142. 10.1016/j.sbi.2017.02.005 28364585

[B73] Nagano S. Poulos T. L. (2005). Crystallographic study on the dioxygen complex of wild-type and mutant cytochrome P450cam:Implications for the dioxygen activation mechanism. J. Biol. Chem. 22102–22107. 10.1074/jbc.M501732200 15994329

[B74] Nagano S. Cupp-Vickery J. R. Poulos T. L. (2005). Crystal structures of the ferrous dioxygen complex of wild-type cytochrome P450eryF and its mutants, A245S and A245T: investigation of the proton transfer system in P450eryF. J. Biol. Chem. 280, 22102–22107. 10.1074/jbc.M501732200 15824115

[B75] Nelson J. W. Subrahmanyan R. M. Summers S. A. Xiao X. S. Alkayed N. J. (2013). Soluble epoxide hydrolase dimerization is required for hydrolase activity. J. Biol. Chem. 288, 7697–7703. 10.1074/jbc.M112.429258 23362272 PMC3597810

[B76] Nisimoto Y. Kinosita K. Jr. Ikegami A. Kawai N. Ichihara I. Shibata Y. (1983). Possible association of NADPH-cytochrome P-450 reductase and cytochrome P-450 in reconstituted phospholipid vesicles. Biochemistry 22, 3586–3594. 10.1021/bi00284a008 6412745

[B77] Operana T. N. Tukey R. H. (2007). Oligomerization of the UDP-glucuronosyltransferase 1A proteins: homo- and heterodimerization analysis by fluorescence resonance energy transfer and co-immunoprecipitation. J. Biol. Chem. 282, 4821–4829. 10.1074/jbc.M609417200 17179145

[B78] Ortiz de Montellano P. R. (2005). Cytochrome P450: Structure, Mechanism, and Biochemistry. 3rd. Edn. New York, NY: Kluwer Academic/Plenum Publishers.

[B79] Pearson J. T. Hill J. J. Swank J. Isoherranen N. Kunze K. L. Atkins W. M. (2006). Surface plasmon resonance analysis of antifungal azoles binding to CYP3A4 with kinetic resolution of multiple binding orientations. Biochemistry 45, 6341–6353. 10.1021/bi0600042 16700545 PMC2701698

[B80] Peng H. M. Liu J. Forsberg S. E. Tran H. T. Anderson S. M. Auchus R. J. (2014). Catalytically relevant electrostatic interactions of cytochrome P450c17 (CYP17A1) and cytochrome b5. J. Biol. Chem. 289, 33838–33849. 10.1074/jbc.M114.608919 25315771 PMC4256320

[B81] Petrotchenko E. V. Pedersen L. C. Borchers C. H. Tomer K. B. Negishi M. (2001). The dimerization motif of cytosolic sulfotransferases. FEBS Lett. 490, 39–43. 10.1016/S0014-5793(01)02129-9 11172807

[B82] Podust L. M. Poulos T. L. Waterman M. R. (2001). Crystal structure of cytochrome P450 14alpha -sterol demethylase (CYP51) from *Mycobacterium tuberculosis* in complex with azole inhibitors. Proc. Natl. Acad. Sci. U.S.A. 98, 3068–3073. 10.1073/pnas.061562898 11248033 PMC30608

[B83] Polekhina G. Board P. G. Blackburn A. C. Parker M. W. (2001). Crystal structure of maleylacetoacetate isomerase/glutathione transferase zeta reveals the molecular basis for its remarkable catalytic promiscuity. Biochemistry 40, 1567–1576. 10.1021/bi002249z 11327815

[B84] Praporski S. Ng S. M. Nguyen A. D. Corbin C. J. Mechler A. Zheng J.. (2009). Organization of cytochrome P450 enzymes involved in sex steroid synthesis: PROTEIN-PROTEIN INTERACTIONS IN LIPID MEMBRANES. J. Biol. Chem. 284, 33224–33232. 10.1074/jbc.M109.006064 19805543 PMC2785165

[B85] Raman S. Lange O. F. Rossi P. Tyka M. Wang X. Aramini J.. (2010). NMR structure determination for larger proteins using backbone-only data. Science 327, 1014–1018. 10.1126/science.1183649 20133520 PMC2909653

[B86] Reed J. R. Backes W. L. (2016). The functional effects of physical interactions involving cytochromes P450: putative mechanisms of action and the extent of these effects in biological membranes. Drug Metab. Rev. 48, 453–469. 10.1080/03602532.2016.1221961 27500687 PMC5108515

[B87] Reed J. R. Backes W. L. (2017). Physical Studies of P450-P450 Interactions: Predicting Quaternary Structures of P450 complexes in membranes from their X-ray crystal structures. Front. Pharmacol. 8:28. 10.3389/fphar.2017.00028 28194112 PMC5276844

[B88] Reed J. R. Eyer M. Backes W. L. (2010). Functional interactions between cytochromes P450 1A2 and 2B4 require both enzymes to reside in the same phospholipid vesicle: evidence for physical complex formation. J. Biol. Chem. 285, 8942–8952. 10.1074/jbc.M109.076885 20071338 PMC2838316

[B89] Rohe H. J. Ahmed I. S. Twist K. E. Craven R. J. (2009). PGRMC1 (progesterone receptor membrane component 1): a targetable protein with multiple functions in steroid signaling, P450 activation and drug binding. Pharmacol. Ther. 121, 14–19. 10.1016/j.pharmthera.2008.09.006 18992768 PMC2659782

[B90] Ryu C. S. Klein K. Zanger U. M. (2017). Membrane associated progesterone receptors: promiscuous proteins with Pleiotropic Functions - Focus on Interactions with Cytochromes P450. Front. Pharmacol. 8:159. 10.3389/fphar.2017.00159 28396637 PMC5366339

[B91] Schoch G. A. Yano J. K. Sansen S. Dansette P. M. Stout C. D. Johnson E. F. (2008). Determinants of cytochrome P450 2C8 substrate binding: structures of complexes with montelukast, troglitazone, felodipine, and 9-cis-retinoic acid. J. Biol. Chem. 283, 17227–17237. 10.1074/jbc.M802180200 18413310 PMC2427337

[B92] Schwarz D. Kruger V. Chernogolov A. A. Usanov S. A. Stier A. (1993). Rotation of cytochrome P450SCC (CYP11A1) in proteoliposomes studied by delayed fluorescence depolarization. Biochem. Biophys. Res. Commun. 195, 889–896. 10.1006/bbrc.1993.2128 8373424

[B93] Schwarze W. Bernhardt R. Janig G. R. Ruckpaul K. (1983). Fluorescent energy transfer measurements on fluorescein isothiocyanate modified cytochrome P-450 LM2. Biochem. Biophys. Res. Commun. 113, 353–360. 10.1016/0006-291X(83)90473-4 6407482

[B94] Scott E. E. White M. A. He Y. A. Johnson E. F. Stout C. D. Halpert J. R. (2004). Structure of mammalian cytochrome P450 2B4 complexed with 4-(4-chlorophenyl)imidazole at 1.9 {angstrom} resolution: insight into the range of P450 conformations and coordination of redox partner binding. J. Biol. Chem. 279, 27294–27301. 10.1074/jbc.M403349200 15100217

[B95] Sevrioukova I. F. Li H. Zhang H. Peterson J. A. Poulos T. L. (1999). Structure of a cytochrome P450-redox partner electron-transfer complex. Proc. Natl. Acad. Sci. U.S.A. 96, 1863–1868. 10.1073/pnas.96.5.1863 10051560 PMC26702

[B96] Sevrioukova I. Truan G. Peterson J. A. (1997). Reconstitution of the fatty acid hydroxylase activity of cytochrome P450BM-3 utilizing its functional domains. Arch. Biochem. Biophys. 340, 231–238. 10.1006/abbi.1997.9895 9143326

[B97] Shimada T. Mernaugh R. L. Guengerich F. P. (2005). Interactions of mammalian cytochrome P450, NADPH-cytochrome P450 reductase, and cytochrome b(5) enzymes. Arch. Biochem. Biophys. 435, 207–216. 10.1016/j.abb.2004.12.008 15680923

[B98] Simonov A. N. Holien J. K. Yeung J. C. Nguyen A. D. Corbin C. J. Zheng J.. (2015). Mechanistic Scrutiny Identifies a Kinetic Role for Cytochrome b5 Regulation of Human Cytochrome P450c17 (CYP17A1, P450 17A1). PLoS ONE 10:e0141252. 10.1371/journal.pone.0141252 26587646 PMC4654539

[B99] Spera R. Festa F. Bragazzi N. L. Pechkova E. LaBaer J. Nicolini C. (2013). Conductometric monitoring of protein-protein interactions. J. Proteome Res. 12, 5535–5547. 10.1021/pr400445v 24106799

[B100] Stayton P. S. Fisher M. T. Sligar S. G. (1988). Determination of cytochrome b5 association reactions. characterization of metmyoglobin and cytochrome P-450cam binding to genetically engineered cytochromeb5. J. Biol. Chem. 263, 13544–13548. 3417673

[B101] Sugishima M. Sato H. Higashimoto Y. Harada J. Wada K. Fukuyama K.. (2014). Structural basis for the electron transfer from an open form of NADPH-cytochrome P450 oxidoreductase to heme oxygenase. Proc. Natl. Acad. Sci. U.S.A. 111, 2524–2529. 10.1073/pnas.1322034111 24550278 PMC3932878

[B102] Sulc M. Hudecek J. Stiborova M. Hodek P. (2008). Structural analysis of binding of a diamantoid substrate to cytochrome P450 2B4: possible role of Arg 133 in modulation of function and activity of this enzyme. Neuro Endocrinol. Lett. 29, 722–727. 18987581

[B103] Suzuki M. Hirata M. Takagi M. Watanabe T. Iguchi T. Koiwai K.. (2014). Truncated UDP-glucuronosyltransferase (UGT) from a Crigler-Najjar syndrome type II patient colocalizes with intact UGT in the endoplasmic reticulum. J. Hum. Genet. 59, 158–162. 10.1038/jhg.2013.138 24401909 PMC3973126

[B104] Szczesna-Skorupa E. Mallah B. Kemper B. (2003). Fluorescence resonance energy transfer analysis of cytochromes P450 2C2 and 2E1 molecular interactions in living cells. J. Biol. Chem. 278, 31269–31276. 10.1074/jbc.M301489200 12766165

[B105] Tripathi S. Li H. Poulos T. L. (2013). Structural basis for effector control and redox partner recognition in cytochrome P450. Science 340, 1227–1230. 10.1126/science.1235797 23744947

[B106] Vallurupalli P. Hansen D. F. Kay L. E. (2008). Structures of invisible, excited protein states by relaxation dispersion NMR spectroscopy. Proc. Natl. Acad. Sci. U.S.A. 105, 11766–11771. 10.1073/pnas.0804221105 18701719 PMC2575287

[B107] Vincent B. Morellet N. Fatemi F. Aigrain L. Truan G. Guittet E.. (2012). The closed and compact domain organization of the 70-kDa human cytochrome P450 reductase in its oxidized state as revealed by NMR. J. Mol. Biol. 420, 296–309. 10.1016/j.jmb.2012.03.022 22543241

[B108] Wang J. Edmondson D. E. (2007). Do monomeric vs dimeric forms of MAO-A make a difference? A direct comparison of the catalytic properties of rat and human MAO-A's. J. Neural. Transm. 114, 721–724. 10.1007/s00702-007-0678-8 17401534

[B109] Wang R. W. Bird A. W. Newton D. J. Lu A. Y. Atkins W. M. (1993). Fluorescence characterization of Trp 21 in rat glutathione S-transferase 1-1: microconformational changes induced by S-hexyl glutathione. Protein Sci. 2, 2085–2094. 10.1002/pro.5560021209 8298458 PMC2142333

[B110] Wang T. Arifoglu P. Ronai Z. Tew K. D. (2001). Glutathione S-transferase P1-1 (GSTP1-1) inhibits c-Jun N-terminal kinase (JNK1) signaling through interaction with the C terminus. J. Biol. Chem. 276, 20999–21003. 10.1074/jbc.M101355200 11279197

[B111] Weber G. Farris F. J. (1979). Synthesis and spectral properties of a hydrophobic fluorescent probe: 6-propionyl-2-(dimethylamino)naphthalene. Biochemistry 18, 3075–3078. 10.1021/bi00581a025 465454

[B112] Wen B. Doneanu C. E. Lampe J. N. Roberts A. G. Atkins W. M. Nelson S. D. (2005). Probing the CYP3A4 active site by cysteine scanning mutagenesis and photoaffinity labeling. Arch. Biochem. Biophys. 444, 100–111. 10.1016/j.abb.2005.09.010 16289363

[B113] Wen B. Lampe J. N. Roberts A. G. Atkins W. M. David Rodrigues A. Nelson S. D. (2006). Cysteine 98 in CYP3A4 contributes to conformational integrity required for P450 interaction with CYP reductase. Arch. Biochem. Biophys. 454, 42–54. 10.1016/j.abb.2006.08.003 16959210 PMC2001172

[B114] Wienkers L. C. Heath T. G. (2005). Predicting *in vivo* drug interactions from *in vitro* drug discovery data. Nat. Rev. Drug Discov. 4, 825–833. 10.1038/nrd1851 16224454

[B115] Wilderman P. R. Gay S. C. Jang H. H. Zhang Q. Stout C. D. Halpert J. R. (2012). Investigation by site-directed mutagenesis of the role of cytochrome P450 2B4 non-active-site residues in protein-ligand interactions based on crystal structures of the ligand-bound enzyme. FEBS J. 279, 1607–1620. 10.1111/j.1742-4658.2011.08411.x 22051155 PMC3911823

[B116] Williams P. A. Cosme J. Ward A. Angove H. C. Matak Vinkovic D. Jhoti H. (2003). Crystal structure of human cytochrome P450 2C9 with bound warfarin. Nature. 424, 464–468 10.1038/nature01862 12861225

[B117] Wu E. S. Yang C. S. (1984). Lateral diffusion of cytochrome P-450 in phospholipid bilayers. Biochemistry 23, 28–33. 10.1021/bi00296a005 6691964

[B118] Yablokov E. Florinskaya A. Medvedev A. Sergeev G. Strushkevich N. Luschik A.. (2017). Thermodynamics of interactions between mammalian cytochromes P450 and b5. Arch. Biochem. Biophys. 619, 10–15. 10.1016/j.abb.2017.02.006 28238672

[B119] Yano J. K. Wester M. R. Schoch G. A. Griffin K. J. Stout C. D. Johnson E. F. (2004). The structure of human microsomal cytochrome P450 3A4 determined by X-ray crystallography to 2.05 Å resolution. J. Biol. Chem. 279, 38091–38094. 10.1074/jbc.C400293200 15258162

[B120] Yantsevich A. V. Gilep A. A. Usanov S. A. (2009). Conformational stability of cytochrome b5, enhanced green fluorescent protein, and their fusion protein Hmwb5-EGFP. Biochem. Mosc. 74, 518–527. 10.1134/S000629790905006X 19538125

[B121] Yoshinari K. Petrotchenko E. V. Pedersen L. C. Negishi M. (2001). Crystal structure-based studies of cytosolic sulfotransferase. J. Biochem. Mol. Toxicol. 15, 67–75. 10.1002/jbt.1 11284047

[B122] Yuan L. M. Gao Z. Z. Sun H. Y. Qian S. N. Xiao Y. S. Sun L. L.. (2016). Inter-isoform Hetero-dimerization of Human UDP-Glucuronosyltransferases (UGTs) 1A1, 1A9, and 2B7 and Impacts on Glucuronidation Activity. Sci. Rep. 6:34450. 10.1038/srep34450 27857056 PMC5114717

[B123] Yuan L. Qian S. Xiao Y. Sun H. Zeng S. (2015). Homo- and hetero-dimerization of human UDP-glucuronosyltransferase 2B7 (UGT2B7) wild type and its allelic variants affect zidovudine glucuronidation activity. Biochem. Pharmacol. 95, 58–70. 10.1016/j.bcp.2015.03.002 25770680

[B124] Zhang M. Huang R. Ackermann R. Im S. C. Waskell L. Schwendeman A.. (2016). Reconstitution of the Cytb5-CytP450 Complex in Nanodiscs for Structural Studies using NMR Spectroscopy. Angew. Chem. Int. Ed Engl. 55, 4497–4499. 10.1002/anie.201600073 26924779

